# Temporal and Spatial Dynamics of Anthracnose ( *Colletotrichum sublineolum*) Disease on Selected Sorghum Genotypes at Assosa Zone, Western Ethiopia

**DOI:** 10.12688/f1000research.156145.2

**Published:** 2025-03-17

**Authors:** Binyam Tsedaley, Kumlachew Alemu

**Affiliations:** 1 College of Agriculture and Natural Resource, Assosa University, Assosa, NA, NA, Ethiopia

**Keywords:** Assosa, Bambasi, C. sublineolum, Disease progress rate, Sorghum, Weather variables

## Abstract

**Background:**

Over 500 million people worldwide rely on sorghum as a main food crop. About 10% of the daily caloric intake of households in Ethiopia’s northwest and eastern regions, including Benishangul Gumuz, comes from sorghum. A hemi-biotrophic fungal pathogen called *Colletotrichum sublineolum* caused anthracnose disease is among the biotic constraints of sorghum production.

**Methods:**

Ten selected sorghum genotypes were assessed for sorghum anthracnose severity and its temporal and spatial dynamics on field plots in the districts of Assosa and Bambasi. The performance of the chosen sorghum genotypes was assessed using the following metrics: AUDPC, disease progress rate, yield-related trait, sorghum grain yield, and mean severity index. Anthracnose severity was evaluated using a 1–5 disease rating scale and assessments conducted at seven consecutive time points.

**Results:**

The result found that the mean anthracnose severity index ranging from 60-77 PSI and 53-82 PSI, respectively. AUDPC varied from 351 to 470 % days and 316 to 499 % days at Assosa and Bambasi districts, respectively. Bambasi district achieved a larger grain yield than the Assosa district. Assosa-1 demonstrated a significant level of disease pressure, yet the current investigation found that this genotype is the highest performing genotype in both locations.

**Conclusions:**

There is a considerable positive link between the severity of anthracnose and the weekly total rainfall and relative humidity. At both trial sites, Mersa-1 continuously produced higher grain yields and reduced disease levels. Breeders might utilize the Baco Striga sorghum genotype as a check line in a breeding effort to resist anthracnose disease because it shown a high vulnerability to the disease at both locations.

## Introduction

Sorghum ( *Sorghum bicolor* L. Moench) is a tropical plant belonging to the *Poacaea* family originated in northeast Africa ( Pedersen et al. 2003). Due to the highest genetic diversity found in the nation for both cultivated and wild varieties, Ethiopia is frequently recognized as the center origin and diversity of sorghum domestication ( Fetene et al. 2011). After maize, rice, wheat, and barley, sorghum is the fifth most important cereal crop in the world ( FAO 2021). Sorghum, which is grown yearly on over 1.8 million hectares of land and contributes 4.3 million t to Ethiopia’s annual grain production, is the third most important cereal crop in the country after maize and teff ( CSA 2017). According to Upadhyaya et al. (2013), sorghum is a crop with several uses, since humans can consume its grains, and its stalks may be utilized to make juice, animal feeds, and construction. Ethiopians typically use sorghum to make injera, which is a traditional bread ( Wortmann et al. 2009). Over 500 million people worldwide, residing in over 30 nations, rely on sorghum as a staple food ( Smith and Frederiksen 2000). About 10% of the daily caloric intake of households in Ethiopia’s northwest and eastern regions, including Benishangul Gumuz, comes from sorghum ( USAID 2012). It is also known to withstand hostile environmental circumstances. However, both biotic and abiotic constraints negatively affect its production.

A hemi-biotrophic fungal pathogen called *Colletotrichum sublineolum* caused anthracnose disease is among the biotic constraints of sorghum production. It limits grain production and quality in most sorghum growing areas in the world, including Ethiopia ( Chala et al. 2007; Tsedaley et al. 2016, 2021 and 2023). Since its discovery in 1902 in Togo, West Africa ( Sutton, 1980), anthracnose disease on sorghum has been documented in most places where the crop may be successfully farmed worldwide ( Pastor-Corrales and Frederiksen 1980; Thakur and Mathur 2000). In tropical areas where high temperatures and humidity promote *C. sublineolum* development, propagule dissemination, and sporulation, this disease is associated with significant sorghum yield losses ( Pastor-Corrales and Frederiksen 1980). Serious epidemics at farmers’ and research fields occur when the crop reaches its flowering stage, which is a condition that many local land races and improved varieties are susceptible at this stage ( Thomas 1995).

One of the most severe diseases affecting sorghum output globally is anthracnose, which is particularly problematic in warm, humid climates ( Thakur 2007; Upadhyaya et al. 2013). The fungus can infect any of the plant’s aerial parts, including the panicle, stalk, foliage, and grain. Although foliar infection is the most dangerous, it can affect the quality of both the grain and the stalk ( Upadhyaya et al. 2013). Damage from sorghum anthracnose might include foliar damage, stalk rot, peduncle breakage, and grain degeneration ( Pastor-Corrales and Frederiksen 1980). According to Ngugi et al. (2000), *C. sublineolum* epidemics were consistently associated with greater disease levels by the milk stage (v95) and higher final disease levels at crop senescence. In susceptible types, the disease can also result in grain yield losses ranging from 47 to 67% ( Pande et al. 2003; Gwary and Asala 2006); the loss of stalk is less severe, at 23% ( Pande et al. 2003). According to reports from eastern Ethiopia, the disease can cause grain yield losses of between 26 and 35% on vulnerable sorghum genotypes ( Girma et al. 2009).

Farmers with limited resources are not advised to employ fungicide as a disease management strategy for anthracnose due to the financial benefits ( Marley et al. 2004). Biological control with the use of antagonistic organisms such as *Trichoderma* species, *Pseudomonas syringae* and *Pseudomonas chlororaphis* have been extensively used by various researchers across the globe due to their characteristics such as fast competition, parasitism, plant root colonization, induced systemic resistance, plant growth promotion, production of antibiosis ( Khan et al. 2018; Sharma et al. 2019). The primary approach to managing this disease has been genetic resistance; however, due to the great degree of heterogeneity in the pathogen population, host-specific resistance is frequently unstable ( Ali et al. 1987; Bressan and Figueiredo 2003). Sorghum anthracnose disease can be effectively controlled with resistant cultivars. However, because the pathogen population is highly variable and novel pathotypes have the ability to overcome existing sources of resistance, numerous sources of resistance are required ( Cardwell et al. 1989; Valerio et al. 2005). Because, *C. sublineolum* is highly changeable and the environment has a significant impact on the development of symptoms and the transmission of the disease, breeding for stable host plant resistance has proven challenging, especially in areas where anthracnose is prevalent. The underlying mechanisms of anthracnose resistance remain poorly understood, despite the fact that many sources of genetic resistance are identified ( Tesso et al. 2012). Ethiopian sorghum germplasm may have genetic diversity for anthracnose resistance breading, and it has proven to be a useful source of genetic variation for the creation of grain sorghum breeding lines in the United States ( Erpelding 2009).

Sorghum anthracnose is a global sorghum disease that is common anywhere sorghum is grown. It is frequently seen in farmer’s fields in Ethiopia’s main sorghum-growing regions ( Tsedaley et al. 2016). In the western portion of the nation, the majority of native land races, including enhanced sorghum genotypes, are vulnerable to this disease. Sorghum anthracnose is a serious disease in the Assosa zone, especially in the districts of Assosa, Bambasi, and Homesha, according to Tsedaley et al. (2016). Furthermore, the majority of the farmers in that region constantly inquired about increasing the genotype of high-yielding, disease-tolerant sorghum varieties. In the meanwhile, not much study has been done to assess how different sorghum genotypes respond to the anthracnose disease in Benshangul Gumuz, Western Ethiopia. Consequently, the goal of this study was to evaluate the temporal and spatial dynamics of the anthracnose ( *Colletotrichum sublineolum*) disease on several genotypes of sorghum in the Assosa zone of Western Ethiopia.

## Methods

### Explanation of the research areas

During the 2020 main cropping season, field experiments were conducted at two locations, referred to as Assosa district (Assosa University research field) and Bambasi district, to assess how different sorghum genotypes responded to specific disease variations and how they adapted to different agro-ecological zones of the study areas. At a height of 1462 meters above sea level, the Bambasi district experimental site is situated at 9°9′N longitude and 34°66′E latitude. On the other hand, the Assosa district experimental site is situated at an elevation of 1600 meters above sea level and at 34°59′E longitude and 10°10′N latitude ( Tsedaley et al. 2016). Situated in the semi-arid tropical belts of Western Ethiopia, the experimental sites exhibit humid climate conditions.

### Materials for the experiments, preparation of the land, experimental units, and design

**Materials for the experiments**

In the Assosa and Bambasi districts during the main cropping season of 2020, the response of ten sorghum genotypes ( Table 1), with varying degrees of resistance to sorghum anthracnose were assessed.

**Table 1.** Sorghum varieties with their crop passport.

S.N	Variety name	Pedigree name	Year of release	Disease reaction	Altitude	Yield (q/ha)		Seed source
						Research center	Farmers field	
1	Aba-Melko	Sartu	2001	R		75	50	JARC
2	Chemeda	ACC-BRC-18	2013	MR	1500-1900	32	25	BARC
3	Dano	BRC-378	2006	MR	1500-1900	40-50	30-48	BARC
4	Gemedi	ACC-BRC-5	2013	MR	1500-1900	33	28	BARC
5	Lalo	BRC-245	2006	MR	1500-1900	40-52	35-48	BARC
6	Universally Susceptible	BTX623	-	HS	-	-	-	MARC
7	-	PI570726	-	HR ^*^	-	-	-	MARC
8	-	SC748-5	-	HR ^*^	-	-		MARC
9	Assosa-1	-	Under seed multiplication	-	-	-	-	Assosa University
10	Local Chuck	-	-	-	-	-	-	-

Source: MOA and BARC.

Where: HR* = Highly Resistant under greenhouse, HS = Highly Susceptible, MR = Moderately Resistant, R = Resistant, S = Susceptible and - = no information.

**Preparation of the Land**

In each trial location, the grounds were prepped by plowing twice. DAP and urea fertilizers were applied at recommended rates of 100 kg ha ^-1^ ( Mekbib 2006). DAP was applied at the time of the sowing, and urea was added when the sorghum plants were knee-high (0.50 m). Following seed emergence, cultivation and weed management are done three times ( Tsedaley et al. 2023).

**Experimental units and design**

Ten sorghum genotypes that were evaluated in the 2020 cropping season in the districts of Assosa and Bambasi served as the experimental units. In each experimental site, the trials were set up in a randomized complete block design (RCBD) with three replications. According to Tsedaley et al. (2023), the trials were carried out in naturally infected fields without the use of artificial inoculants. Plots and blocks were separated by 1.5 and 1 meters, respectively. There were five rows and 2 m × 3.75 m (7.5 m ^2^) area in each allotment. Inter-row spacing of 0.75 m and intra-row spacing of 0.20 m were used according to Mekbib (2006). Ten sorghum plants per row and fifty plants total were cultivated in each plot. In the districts of Bambasi and Assosa, sowing took place on June 10 and June 11, respectively. Twenty-one days after seeding, plants were trimmed to one plant per hill from two seeds each ( Chala et al. 2010a).

### Data gathering

**Evaluation of Sorghum Anthracnose Disease Severity**

Ten randomly chosen and pre-tagged plants in the middle three rows were used to measure the percentage of their leaf area that was impacted by the anthracnose disease. According to this study, the disease started to manifest in the first week of September in the cropping season of 2020 in both the Assosa and Bambasi areas. Beginning 12 weeks (85 days) following each location’s seeding, the severity of the anthracnose disease was rated at intervals of seven days for seven consecutive time points. It began when the Baco Striga and BTx623 (universally susceptible) sorghum genotypes at both sites began to exhibit definite disease symptoms. It was evaluated using the Thakur et al. (2007) recommended 1–5 disease rating scale. For analytical purposes, the sorghum anthracnose severity grade was transformed into a percent severity index (PSI) using the Wheeler (1969) formula:

$$\mathrm{PSI}=\frac{\mathrm{Snr}}{\mathrm{Npr}\times \mathrm{Mss}}\times 100$$

where Mss is the maximum disease scale, Npr is the number of plants assessed, and Snr is the sum of numerical ratings.

In order to compute disease progress rates (r), one must use the linearized logistic model ( Van der Plank 1963) and evaluate the resultant number using SAS software.

$$r=\frac{\left(\ln\frac{X}{1-X}\right)-\left(\ln\frac{X_{o}}{1-X_{0}}\right)}{t}$$

Where t is the length of the epidemic, r is the disease progression rate, Xo is the starting disease severity, X is the end disease severity, and ln is the natural logarithm.

The percent severity index (PSI) data was used to determine the area under the disease progress curve (AUDPC), which is done using the technique suggested by Shaner and Finney in 1977.

$$\text{AUDPC}=\sum_{i=1}^{n-1}\left[\frac{\left(X_{i}+X_{i+1}\right)}{2}\right]\left(t_{i+1}-t_{1}\right)$$

Where n is the total number of disease assessments, t _i_ is the period in days since the initial assessment date, and Xi is the percentage of leaf area covered by anthracnose at the i ^th^ observation.

**Sorghum agronomic data, yield, and yield-related characteristics**

Performance in terms of plant height, panicle length, 1000-seed weight (TSW), and grain yield was also assessed for sorghum genotypes. Ten randomly chosen and tagged plants in the middle three rows were counted to determine the number of leaves on each plant at the maturity stage. Plant height is determined by taking a measurement from the top of the ground to the tips of the panicles of the ten plants in each of the middle three rows. Measurement of the length of each panicle from base to tip was used to record the length of each panicle. At harvest, the weight of 1000 seeds and the grain yield (kg/plot) were recorded from the plants in the middle three rows of each plot. From harvested regions, the grain yield was calculated. Last but not least, the grain yield per hectare (t ha ^−1^) was computed by translating the grain yield from each plot’s middle three harvested rows into hectares. The grains’ moisture content was changed to 12.5% ( Tsedaley et al. 2023).

### Data analysis

All disease assessment and agronomic data were analyzed using SAS software version 9.4 ( SAS 2015). LSD test at p<0.01 level of probability was used for mean comparisons among treatments for treatments with significant differences. Correlation tests were carried out between disease parameters and grain yield, weather variables and disease severity, disease severity and grain yield, and AUDPC with grain yield, as well as yield-related traits of the tested sorghum genotypes in the two districts, and analyzed using SAS software. Regression analysis between AUDPC and grain yield was performed using SAS software version 9.4.

## Results

### Sorghum genotype responses to anthracnose disease

At both sites (Assosa and Bambasi) districts, the mean disease severity of all evaluated sorghum genotypes differed significantly (p<0.01 and p<0.001), respectively. The average severity of anthracnose in the Assosa district ranged from 59.6 to 77.3 PSI. The genotype of Baco Striga sorghum was found to be sensitive (77.3 PSI), whereas the most resistant genotype was Mersa-1 (59.6 PSI), followed by SC748-5 (66.8 PSI). The mean severity of anthracnose in the Bambasi district ranged from 52.8 to 82.4 PSI ( Table 2). Similarly, the ACC-BRC-18 genotype was the most resistant (52.8 PSI) compared to the other genotypes. Whereas, Baco Striga sorghum genotype which was shown to be sensitive (82.4 PSI). Across the seven sorghum genotypes that were evaluated, at Assosa district had a higher overall disease pressure than Bambasi district. This implied that MAS decreases between 1 and 19 PSI were seen. This showed that the seven evaluated sorghum genotypes growing in the Bambasi district had MAS reductions ranging from 1 to 19 PSI. At Bambasi district ACC-BRC-18 and ACC-BRC-5 genotypes had the lowest anthracnose severity score as compared with others.

**Table 2.** Sorghum genotype responses to anthracnose severity index and rate of disease progression at Assosa and Bambasi districts, Ethiopia.

Genotype	Mean anthracnose severity (PSI)		Disease progress rate (logit day ^−1^)	
	Assosa	Bambasi	Assosa	Bambasi
Baco Striga	77.3 ± 7.6 ^a^	82.4 ± 2.4 ^a^	0.195 ± 0.003 ^a^	0.184 ± 0.003 ^a^
ACC-BRC-18	71.5 ± 8.1 ^abc^	52.8 ± 3.7 ^g^	0.070 ± 0.014 ^b^	0.027 ± 0.008 ^ef^
BRC-378	74.9 ± 5.7 ^ab^	58.5 ± 4.8 ^efg^	0.079 ± 0.027 ^b^	0.031 ± 0.011 ^ef^
ACC-BRC-5	73.4 ± 10.4 ^abc^	55.8 ± 3.5 ^fg^	0.071 ± 0.027 ^b^	0.021 ± 0.004 ^f^
BRC-245	73.5 ± 2.0 ^abc^	61.7 ± 4.5 ^de^	0.072 ± 0.025 ^b^	0.039 ± 0.010 ^de^
BTx623	74.2 ± 2.2 ^abc^	63.8 ± 6.3 ^cde^	0.061 ± 0.004 ^b^	0.059 ± 0.018 ^bc^
Mersa-1	59.6 ± 2.3 ^d^	70.9 ± 2.8 ^b^	0.056 ± 0.021 ^b^	0.061 ± 0.005 ^b^
SC748-5	66.0± 9.3 ^cd^	68.2 ± 2.6 ^bc^	0.073 ± 0.016 ^b^	0.193 ± 0.004 ^a^
Geremew	66.7± 7.0 ^bcd^	65.5 ± 1.0 ^bcd^	0.054 ± 0.010 ^b^	0.048 ± 0.011 ^cd^
Assosa-1	74.7± 1.4 ^ab^	60.3± 1.7 ^def^	0.063 ± 0.021 ^b^	0.025 ± 0.004 ^f^
CV (%)	7.03	5.2	20.91	10.4

Mean values with the same superscript characters do not differ substantially from one another at p<0.01%, CV stands for coefficient of variation.

In the 2020 cropping season, the disease progress rate was calculated for all sorghum genotypes at the two districts (Assosa and Bambasi). Among the tested sorghum genotypes, there was a highly significant (p<0.001) difference in the observed disease rate ( Table 2). The disease progress rate at Assosa district ranged from 0.054 to 0.195 (logit day ^−1^). The Baco Striga showed the greatest rate of disease development (0.195), whilst the Geremew genotype showed the slowest rate of disease development (0.054) compared to other genotypes. The disease progression rate in the Bambasi district ranged from 0.021 to 0.193. The SC748-5 and Baco Striga genotypes showed a quicker rate of disease progress (0.193 and 0.184, respectively). On ACC-BRC-5 sorghum genotypes, the slowest disease development (0.021) was observed in the Bambasi district. All studied sorghum genotypes, with the exception of Baco Striga and SC748-5 genotypes planted in the Bambasi district as opposed to the Assosa district, showed decreased rates of disease development overall. In general, Assosa district saw a faster rate of disease development than Bambasi district for the genotypes of sorghum that were evaluated.

**Figure 1.**
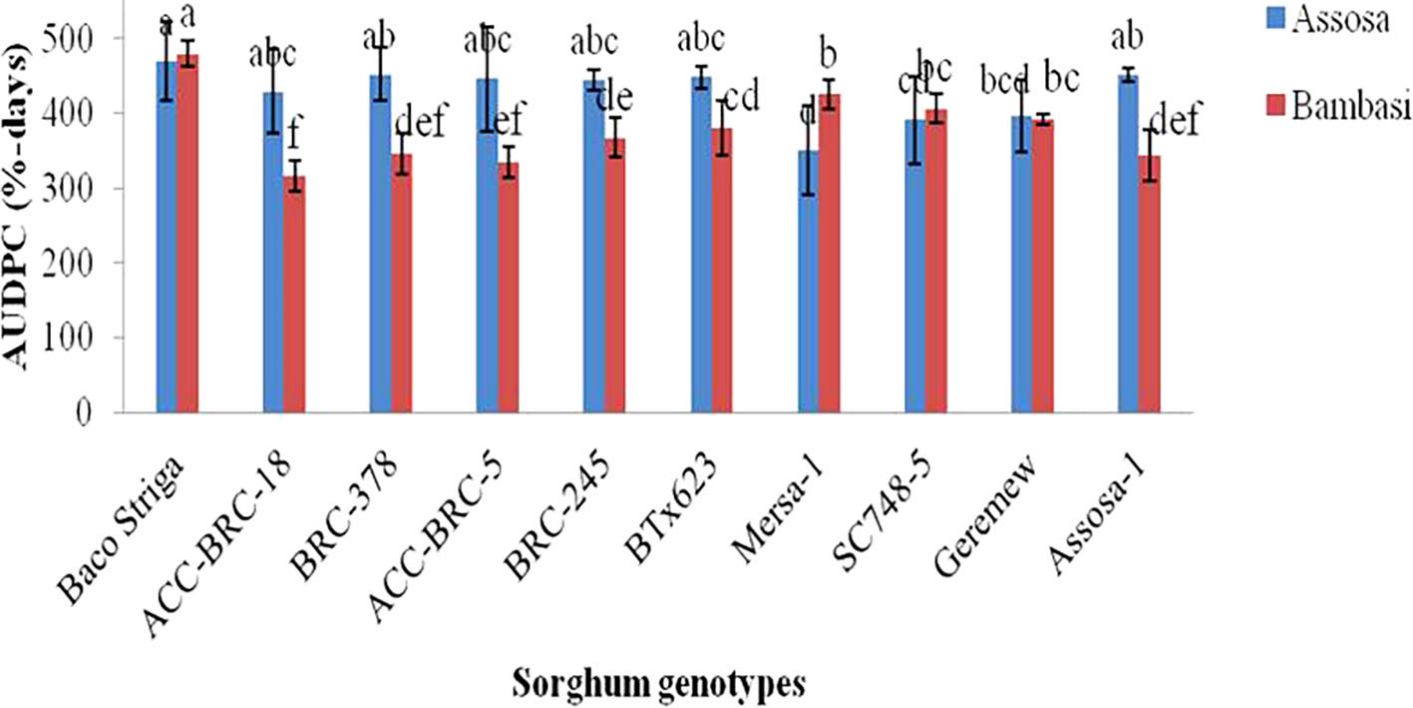
AUDPC of anthracnose disease on ten sorghum genotypes examined in Assosa and Bambasi districts, Ethiopia.

The AUDPC analysis, which was conducted using severity records, revealed substantial (p<0.01 and p<0.001) variations in the genotypes of sorghum examined across the two districts (Assosa and Bambasi) in the 2020 cropping season ( Figure 1). The Assosa district’s AUDPC ranged from 351% to 470% of days. The Baco Striga yielded the greatest AUDPC value (470% days), indicating a genotype that was sensitive to the disease. On the other hand, Mersa-1 produced the lowest AUDPC value (351% days), which was followed by genotypes SC748-5 and Geremew, which were found to be resistant ( Figure 1). The AUDPC ranged from 316 to 499% days in the Bambasi district. Baco Striga, the genotype that was released as Striga resistant, has the highest AUDPC score (499% days). Conversely, the genotypes ACC-BRC-18 and ACC-BRC-5, which were discovered to be resistant genotypes, had the lowest AUDCP values (316 and 334% days) when assessed ( Figure 1). With the exception of Baco Striga, Mersa-1, and SC748-5, all genotypes had greater AUDPC values in the Assosa district compared to the Bambasi district.

### Sorghum anthracnose’s temporal dynamics

**Figure 2.**
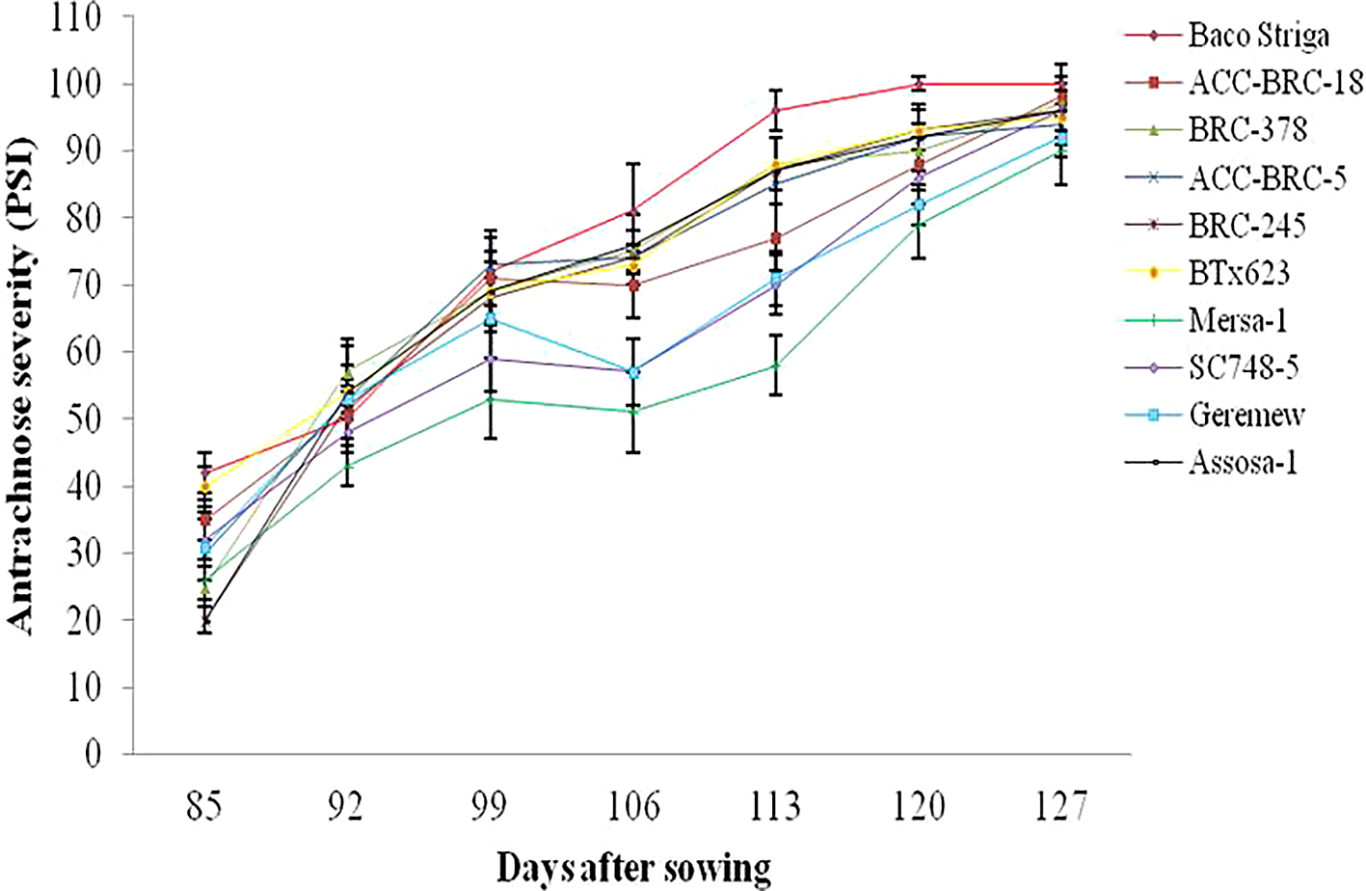
Disease progression curves for anthracnose outbreaks on 10 sorghum genotypes examined in Assosa district, Ethiopia.

**Figure 3.**
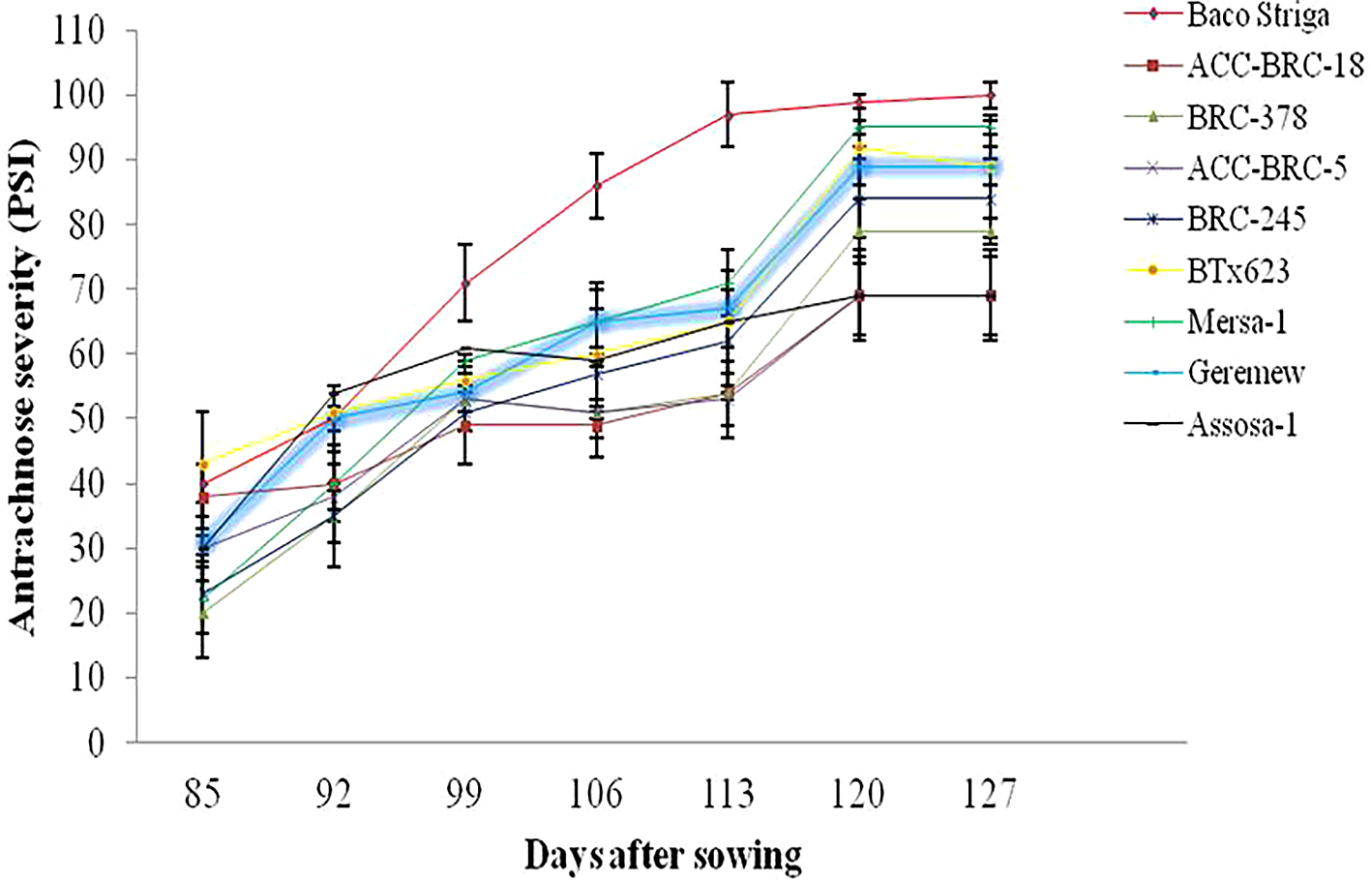
Disease progression curves for anthracnose outbreaks on 10 sorghum genotypes examined in Bambasi district, Ethiopia.

During the 2020 main cropping season, there were differences in the beginning and progression rate of anthracnose disease across the genotypes and sites that sorghum were evaluated. During the 2020 main cropping season, the disease began sooner and spread quickly on Baco Striga sorghum genotypes in both Assosa and Bambasi districts ( Figures 2 and 3). On the other hand, it appeared somewhat later and developed more slowly in Assosa district for the Mersa-1, SC748-5, and Geremew sorghum genotypes, and in Bambasi district for the ACC-BRC-18, ACC-BRC-5, and BRC-245 sorghum genotypes. Seven days’ worth of data revealed a statistically significant rise in the severity of anthracnose on all genotypes of sorghum that were evaluated in the second and third weeks in Assosa district and the second, third, and sixth weeks in Bambasi district. When the plants were in the flowering to grain filling stages, the anthracnose severity was at its maximum across all genotypes evaluated. In both places, this time frame matched the first and second week of October ( Figures 2 and 3).

### Sorghum genotypes’ agronomic characteristics

The agronomic characteristics (Leave number and Plant height) of all genotypes of sorghum that were evaluated demonstrated a highly significant (p<0.001) difference between each other within the two districts (Assosa and Bambasi) ( Table 3). In both locations, Assosa-1 sorghum genotype was grown to a height of 18 leaves. Whereas, the lowest (7) leave number recorded on BTx623 genotype followed by, Baco Striga at both locations. The majority of the sorghum genotypes that were evaluated generally had comparable leaf numbers at both locations. The genotype BRC-245 cultivated at both locations consistently measured the tallest plant height. However, the dwarf plant height on the genotype of BTx623 sorghum growing at both locations was measured. The majority of the sorghum genotypes that were evaluated grew marginally higher in the Assosa district compared to the Bambasi district.

**Table 3.** Effects of sorghum genotypes on plant height and number of leaves in Assosa and Bambasi districts, Ethiopia.

Genotype	Leave number		Plant height (cm)	
	Assosa district	Bambasi district	Assosa district	Bambasi district
Baco Striga	8.1 ± 1.9 ^ef^	6.7 ± 0.2 ^f^	145.7 ± 53.8 ^de^	116.6 ± 13.2 ^de^
ACC-BRC-18	14.7 ± 8.1 ^ab^	15.0 ± 1.2 ^b^	290.9 ± 36.6 ^ab^	294.5 ± 39.6 ^a^
BRC-378	13.3 ± 4.4 ^c^	12.3 ± 1.7 ^c^	295.3 ± 43.5 ^ab^	290.2 ± 30.9 ^a^
ACC-BRC-5	15.3 ± 1.4 ^abc^	13.0 ± 0.9 ^c^	276.0 ± 45.7 ^b^	235.3 ± 40.3 ^b^
BRC-245	13.6 ± 0.7 ^c^	12.5 ± 1.5 ^c^	327.9 ± 35.6 ^a^	322.5 ± 30.5 ^a^
BTx623	6.9 ± 0.2 ^f^	7.3 ± 0.1 ^ef^	85.5 ± 2.8 ^f^	90.9 ± 1.9 ^e^
Mersa-1	11.4 ± 0.2 ^d^	9.4 ± 1.1 ^d^	208.7 ± 17.8 ^c^	208.7 ± 31.5 ^c^
SC748-5	8.8± 0.8 ^e^	7.3± 0.6 ^ef^	121.9 ± 12.7 ^def^	119.8 ± 35.2 ^de^
Geremew	10.9 ± 1.0 ^d^	9.5± 0.5 ^d^	107.6 ± 4.3 ^ef^	110.2 ± 10.2 ^de^
Assosa-1	18.4± 1.7 ^a^	17.5± 2.3 ^a^	142.8 ± 17.7 ^de^	139.9 ± 10.6 ^cd^
CV (%)	7.5	10.8	10.8	13.9

Mean values with the same superscript characters do not differ substantially from one another at p<0.01%, CV stands for coefficient of variation.

### Components of sorghum genotypes related to yield

The yield-related components of all genotypes of sorghum examined shown a highly significant (p<0.01) difference between each other within the two districts (Assosa and Bambasi) ( Table 3). With the exception of Baco Striga and SC748-5, the panicle length of every studied sorghum genotype revealed a taller look in the Bambasi district compared to the Assosa district. The examined genotypes’ grain yields are strongly positively correlated with the taller appearances of the panicle length ( Table 7). However, all examined sorghum genotypes in the Bambasi district showed somewhat higher 1000-seed weight (TSW) than in the Assosa district. In the Bambasi district, this clearly demonstrated increased grain yield increments and substantial positive associations for these genotypes ( Table 3). This demonstrated the degree to which the Bambasi district’s climate was conducive to the production of sorghum and the ability of the plants to assimilate photosynthetically into grains. Grain yields in the Assosa district were lower than in the Bambasi district due to the lower TSW recorded there.

### Sorghum genotypes’ grain yields

There was a highly significant (p<0.001) variation in grain production across the 10 genotypes of sorghum that were examined within the districts of Assosa and Bambasi ( Table 4). Grain yield in the Assosa district ranged from 1.2 to 8.4 t ha ^−1^. On the Assosa-1 genotype, the maximum grain yield (8.4 t ha ^-1^) was recorded. On the Mersa-1 genotype, the second-highest grain production (7.9 t ha ^-1^) was recorded ( Table 4). Conversely, grain yield in the Bambasi district ranged from 1.3 to 8.9 t ha ^-1^. On the Assosa-1 genotype, the maximum grain yield of 8.9 t ha-1 was measured similarly. In the meantime, the districts of Assosa and Bambasi recorded the lowest grain yields, measuring 1.2 and 1.3 t ha ^−1^, respectively, based on the identical Baco Striga genotype ( Table 4). Prior to its release, Baco Striga was a genotype resistant to the weed *Striga hermonithica.* However, this genotype produced the least amount of grain at both locations and was therefore vulnerable to sorghum anthracnose disease.

**Table 4.** Effects of sorghum genotypes on panicle length, TSW, and grain yield in districts of Assosa and Bambasi, Ethiopia.

Genotype	Panicle length (cm)		TSW (g)		Grain yield (t ha ^−1^)	
	Assosa district	Bambasi district	Assosa district	Bambasi district	Assosa district	Bambasi district
Baco Striga	18.5 ± 2.1 ^d^	17.1 ± 2.3 ^d^	14.7 ± 0.6 ^g^	15.7 ± 0.4 ^e^	1.2 ± 0.2 ^f^	1.3 ± 0.3 ^f^
ACC-BRC-18	24.1 ± 0.8 ^bc^	29.3 ± 0.8 ^ab^	19.3 ± 0.2 ^e^	19.5 ± 0.2 ^c^	4.0 ± 0.1 ^d^	4.3 ± 0.3 ^d^
BRC-378	22.0 ± 4.4 ^dc^	27.7 ± 2.0 ^dc^	25.8 ± 0.7 ^b^	26.4 ± 0.7 ^a^	7.7 ± 0.1 ^b^	7.8 ± 0.2 ^b^
ACC-BRC-5	23.7 ± 2.3 ^bc^	29.2 ± 1.2 ^ab^	23.3 ± 1.6 ^cd^	23.9 ± 1.6 ^b^	3.6 ± 0.2 ^d^	3.8 ± 0.4 ^d^
BRC-245	22.7 ± 2.6 ^c^	27.5 ± 2.0 ^abc^	23.3 ± 0.5 ^d^	24.0 ± 0.1 ^b^	7.7 ± 0.4 ^b^	8.0 ± 0.5 ^b^
BTx623	23.6 ± 2.2 ^bc^	26.0 ± 1.0 ^bc^	15.5 ± 1.1 ^fg^	16.5 ± 0.7 ^ed^	2.8 ± 0.1 ^e^	3.0 ± 0.4 ^e^
Mersa-1	27.3 ± 5.4 ^ab^	30.1 ± 3.9 ^a^	28.5 ± 0.7 ^a^	27.1 ± 1.5 ^a^	7.7 ± 0.2 ^b^	7.9 ± 0.2 ^b^
SC748-5	28.3± 1.0 ^a^	25.6± 0.6 ^c^	16.7 ± 0.8 ^f^	17.7 ± 1.0 ^cd^	1.9 ± 0.1 ^f^	2.2 ± 0.2 ^e^
Geremew	23.7± 0.4 ^bc^	29.3± 0.6 ^ab^	25.1 ± 0.04 ^bc^	25.5 ± 0.8 ^ab^	6.2 ± 0.8 ^c^	6.5 ± 1.0 ^c^
Assosa-1	22.5± 2.3 ^dc^	30.4± 1.8 ^a^	25.3 ± 0.9 ^b^	26.0 ± 1.4 ^a^	8.4 ± 0.1 ^a^	8.9 ± 0.3 ^a^
CV (%)	9.8	7.3	3.8	4.6	6.4	9.0

t is for tonnes, Mean values with the same superscript characters do not differ substantially from one another at p<0.01%, CV stands for coefficient of variation.

Overall, the Bambasi district achieved a larger grain yield than the Assosa district. The Assosa-1 genotype showed the most grain yield increase (0.5 t ha ^−1^) in the Bambasi district. In terms of grain yield, this genotype performed best in both locations. Since the Assosa Agricultural Research Center released this genotype, it has been the most adapted to the Assosa zone, including the current study locations. Assosa-1 demonstrated a significant level of disease pressure, yet the current investigation found that this genotype is the highest performing genotype in both locations. The current study assessed the ACC-BRC-5 and Mersa-1 genotypes as the second highest performing genotypes at both the Assosa and Bambasi districts, in addition to the Assosa-1 genotype.

In general, the Bambasi district saw a higher grain output than the Assosa district for all tested sorghum genotype. This might be because of the increased disease pressure in the Assosa district, which led to a reduction in photosynthetic areas and the development of coalescing necrotic lesions that covered the leaf surface. This finding verified that the Bambasi district was not friendly to the growth of the anthracnose disease, but it was highly advantageous for the production of sorghum crops. Apart from Assosa-1, the genotypes with resistance to anthracnose, ACC-BRC-5 and Mersa-1, also demonstrated the best grain yield in both testing locations. These two genotypes also had good quality and the tallest to moderately tall stalks, which produced a high harvestable biomass. Because biomass serves a variety of uses, farmers value this highly.

### Analysis of the relationship between meteorological conditions and the severity of sorghum anthracnose

The ten tested sorghum genotypes were subjected to a correlation coefficient study between the seven round anthracnose severity assessment records and weakly meteorological variables across two locations. The ten sorghum genotypes' anthracnose severity was positively correlated (p<0.01) weekly total rainfall, relative humidity and negatively correlated (p<0.01) with, mean maximum temperature ( Table 5). Nonetheless, a positive but not significant correlation was found between the anthracnose severity and the interaction of the mean minimum and average temperature. According to this finding, mean maximum temperature, relative humidity, and rainfall are more accurate predictors of anthracnose development in the field than mean lowest temperature and average temperature.

**Table 5.** The correlation coefficient between weather variables and the sorghum anthracnose severity (PSI) at Assosa districts in Western Ethiopia, was measured at weekly intervals.

Weather variables	Genotype									
	Baco Striga	ACC-BRC-18	BRC-378	ACC-BRC-5	BRC-245	BTx623	Mersa-1	SC748-5	Geremew	Assosa-1
WTRF	0.64 *	0.72 **	0.68 *	0.69 **	0.75 **	0.74 **	0.47 *	0.54 *	0.54 *	0.78 **
Min To	0.23 ^ns^	0.46 ^ns^	0.45 ^ns^	0.45 ^ns^	-0.35 ^ns^	0.27 ^ns^	0.31 ^ns^	0.35 ^ns^	0.35 ^ns^	-0.06 ^ns^
Max To	-0.23 ^ns^	-0.16 ^ns^	-0.15 ^ns^	-0.14 ^ns^	-0.27 ^ns^	-0.30 ^ns^	-0.09 ^ns^	-0.12 ^ns^	-0.13 ^ns^	-0.63 *
Ave To	0.37 ^ns^	0.18 ^ns^	0.20 ^ns^	0.17 ^ns^	0.34 ^ns^	0.41 ^ns^	0.23 ^ns^	0.27 ^ns^	0.27 ^ns^	0.66 ^ns^
RH	0.63 *	0.83 **	0.80 **	0.80 **	0.81 ^**^	0.76 **	0.54 *	0.65 *	0.65 *	0.73 **

WTRF: Weekly total rainfall, Min To: minimum temperature, Max To: Maximum temperature, Ave To: Average temperature, RH: Relative Humidity; ns: Not important.

*** Significant at *p*<0.001.

** Significant at *p*<0.01.

* Significant at *p*<0.05.

### Correlation study between the severity and rate of disease progression of the sorghum anthracnose disease and the grain yield of the crop

The analysis of the correlation coefficient between the grain yield of ten sorghum genotypes grown in the Assosa and Bambasi districts and the records of the seven rounds of anthracnose severity assessment revealed a negative non-significant relationship from the first to the fifth round of assessments, and a negative significant (p<0.05) relationship at the sixth and seventh round of assessments ( Table 6).

**Table 6.** The correlation coefficients between the weekly severity of anthracnose and the rate at which the disease progressed and the grain yield of the sorghum genotypes evaluated in the Assosa and Bambasi districts during the 2020 cropping season in the Assosa Zone, Western Ethiopia.

Grain yield tonnes ha ^−1^		Anthracnose severity at days after sowing (PSI)						Disease progression rate
	85	92	99	106	123	130	137	
Assosa	-0.06 ^ns^	-0.06 ^ns^	-0.12 ^ns^	-0.18 ^ns^	-0.24 ^ns^	-0.31 *	-0.23 *	-0.74 ***
Bambasi	-0. 17 ^ns^	-0.05 ^ns^	-0.2 ^ns^	-0.33 ^ns^	-0.40 *	-0.39 *	-0. 36 *	-0.67 ***

PSI = Percent severity index; ns: Not important.

* Significant at *p*<0.05.

** Significant at *p*<0.01.

*** Significant at *p*<0.001.

This finding suggests that when the plant reaches maturity, the disease’s impacts are mostly felt in the reduction of grain output. Furthermore, for the studied sorghum genotypes at both locations, a significant negative correlation was found between the rate of disease progression and grain yield ( Table 4).

### Analysis of the correlation between AUDPC, agronomic factors, grain yield of sorghum, and components linked to yield

In the districts of Assosa and Bambasi, the agronomic characteristics (plant height and leaf number) exhibited strong negative significant (p<0.001) and negative non-significant relationships with AUDPC, respectively ( Table 7). In both locations, there was a strong negative significant (p<0.001) association between panical length and AUDPC. At both locations, TSW displayed a non-significant negative connection with grain yield. Conversely, Table 7 indicates that there was a significant positive association (p<0.01) between the genotypes of sorghum at both locations and the number of leaves, plant height, panicle length, and TSW. This demonstrated the extent to which the disease affected the sorghum genotypes’ grain filling at both locations negatively. Furthermore, the degree of anthracnose damages in sorghum production in these areas was demonstrated by the negative correlation found between AUDPC, agronomic characteristics, grain yield, and yield-related features.

**Table 7.** Sorghum anthracnose (AUDPC) correlation coefficients, agronomic parameters, grain yield, and yield-related components of sorghum genotypes investigated at Assosa and Bambasi districts, Ethiopia.

	Location									
	Assosa district					Bambasi district				
	Leave number	Plant height (cm)	Panicle length (cm)	TSW (gm)	Grain yield t ha ^−1^	Leave number	Plant height (cm)	Panicle length (cm)	TSW (g)	Grain yield t ha ^−1^
AUDPC (%-days)	-0.24 ^ns^	-0.22 ^ns^	-0.69 ***	-0.24 ^ns^	-0.25 *	-0.65 ***	0.51 **	-0.64 ***	-0.33 ^ns^	-0.38 *
Leave number	1	0.56 ***	0.20 ^ns^	0.51 ***	0.56 ***	1	0.57 ***	0.59 ***	0.50 **	0.58 ***
Plant height (cm)		1	0.12 ^ns^	0.33 *	0.36 **		1	0.32 ^ns^	0.33 ^ns^	0.0.40 *
Panicle length (cm)			1	0.33 *	0.33 *			1	0.63 ***	0.62 ***
TSW (g)				1	0.88 ***				1	0.88 ***

AUDPC= area under the disease progress curve, TSW= 1000-seed weight, t = tonnes; ns: Not important.

*** Significant at *p*<0.001.

** Significant at *p*<0.01.

* Significant at *p*<0.05.

### Analysis of regressions between sorghum crop yield and the AUDPC of anthracnose

**Figure 4.**
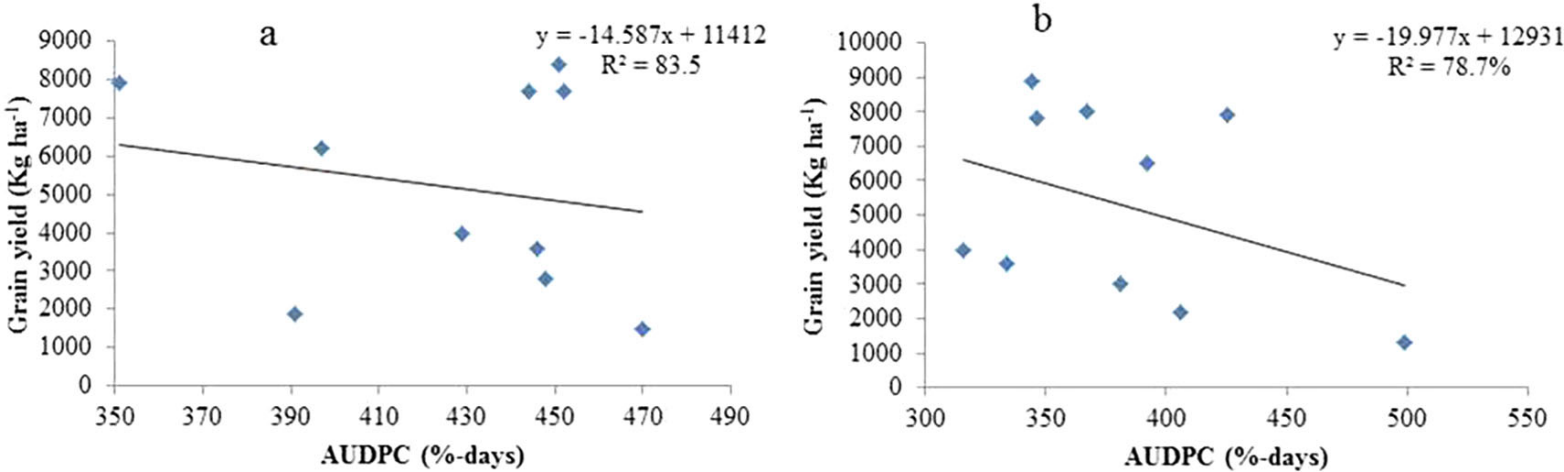
Linear regression relating AUDPC of anthracnose with grain yield on ten sorghum genotypes examined at Assosa (a) and Bambasi (b) districts.

For the ten sorghum genotypes examined in the Assosa and Bambasi districts in the 2020 cropping year, linear regression of the AUDPC was utilized to estimate the grain yield loss ( Figure 4a and b, respectively). Compared to severity linear regression, AUDPC linear regression provided a clearer picture of the link between disease and yield loss. However, disease progress curves are not a reliable indicator of the correlation between disease severity and yield because they are extremely susceptible to changes in epidemiological parameters during the course of the disease. Since the intensity and duration of the disease affect crop production loss, the AUDPC takes these parameters into consideration ( Chaube and Pundhir 2005). This is the association that Sahile et al. (2008) observed in single and mixed cropping systems under Ethiopian conditions between yield loss and chocolate spot on faba beans ( *Vicia faba* L.).

In the cropping season of 2020, the model’s association between anthracnose AUDPC and grain yield explained 83.5 and 78.7% of the variation at Assosa and Bambasi Districts, respectively ( Figure 4a and b). The model-indicated connection between anthracnose AUDPC and grain yield explained 83.5 and 78.7% of the variance at the Assosa and Bambasi Districts ( Figure 4a and b), respectively. With estimated regression coefficients of b1= -20.0 and -14.6 kg ha ^−1^, respectively, the regression line found for Bambasi District had a higher slope than that of Assosa District. The calculations show that on different sorghum genotypes in Bambasi District and Assosa District, there were 20.0 and 14.6 kg ha ^−1^ grain production losses for every percent per day AUDPC increase of sorghum anthracnose, respectively.

## Discussion

Sorghum is the fifth-most important cereal crop in the world, and anthracnose has a significant impact on its productivity. Farmers with limited resources are not advised to employ fungicides as a disease control strategy for anthracnose due to the financial benefits ( Marley et al. 2004). The primary strategy for managing this disease has been genetic resistance; however, due to the great degree of heterogeneity in the pathogen population, host-specific resistance is frequently unstable ( Ali et al. 1987; Bressan and Figueiredo 2003). According to the current study, Assosa district had a higher disease pressure than Bambasi district, and different sorghum genotypes responded to anthracnose disease in different ways at different places. This may be caused by variations in the pathogen’s virulence and aggression as well as local climatic conditions. Additionally, Tsedaley et al. (2021) reported that the isolate from Assosa field 7 (CsAs7), which was tested in a greenhouse setting in Jimma, Ethiopia, on ten known sorghum differentials, was more aggressive and produced the highest severity of anthracnose disease when compared to the isolate obtained from the other major sorghum-growing areas of southwestern and western Ethiopia. As a result, the Assosa district *C. sublineolum* isolate may be crucial to the global development of anthracnose disease-resistant sorghum genotypes through disease resistance screening.

Furthermore, the development of resistant varieties through resistance breeding programs may be significantly influenced by the variations in responses of sorghum genotypes between sites. One of the best and most effective ways to handle sorghum anthracnose in the field is to use resistant cultivars ( Tsedaley et al. 2023). A notable decrease in intensity was noted in the Bambasi district. Moreover, during the main cropping season of 2020, anthracnose severity across the two locations exhibited a non-significant positive correlation with mean minimum temperature and average temperature, a non-significant positive correlation with mean maximum temperature, and a significant positive correlation with weekly total rainfall and relative humidity. Additionally, during the 2014 cropping season, Rana et al. (2021) found a negative correlation between the percent severity index of anthracnose and the highest temperature, minimum temperature, and nighttime relative humidity. Furthermore, Tsedaley et al. (2023) found that, on six frequently tested sorghum genotypes at Jimma during the 2014 and 2015 cropping seasons, anthracnose severity was negatively correlated with mean minimum and mean maximum temperature and positively correlated with weekly total rainfall and average temperature. The current study’s findings suggest that mean maximum temperature, relative humidity, and rainfall are reliable indicators of anthracnose development in the field than mean minimum and average temperature. The percentage severity index of sorghum anthracnose, as reported by Tsedaley et al. (2016), also demonstrated a strong negative correlation with varying altitude ranges. This finding may be related to variations in temperature and relative humidity across various altitudes in western and southwestern Ethiopia. Significant losses are associated with this disease in tropical regions where high temperatures and humidity support the growth, propagule dissemination, and sporulation of *C. sublineolum* ( Pastor-Corrales and Frederiksen 1980).

The current study reveals that the Baco Striga sorghum genotype, which was previously identified as resistant to Striga hermonithica weed, is really very susceptible to anthracnose disease, as seen by the low grain production at both locations. This demonstrated that the breeders disregarded the genotype’s reaction to anthracnose disease, either when they released it or possibly as a result of the presence of various physiological races of C. sublineolum, the pathogen’s variable nature, and its capacity to disrupt the genotypes’ resistant gene. According to Da Costa et al. (2003), C. sublineolum is regarded as a very diverse species due in part to the several pathotypes that have been identified based on varying degrees of virulence on host lines. Furthermore, Prom et al. (2009) noted that the pathogen’s unpredictable character makes it difficult to successfully deploy current cultivars and breed for resistance. As a result, controlling host resistance genes, altering the host-pathogen environment, and lowering the sources of inoculum are essential for the long-term control of anthracnose ( Frederiksen 1986). We verified the unique disease outbreaks observed in these research regions. This could be because of the warm, humid climate in these research sites, which has aided in the disease’s quick onset and spread throughout these regions. Within the two locations, we also saw significant differences in the severity of anthracnose between the genotypes of sorghum that were evaluated. These variations could be caused by changes in the host’s physiological stage, interactions with pathogens, or environmental factors ( Thakur and Mathur 2000). Our data are consistent with the findings of Thakur and Mathur (2000), who found that the pathogen could infect all aboveground plant parts, such as the stalk, leaves, panicle, inflorescence, and grain/seed, reducing the number and quality of both the stalk and the grain. In the current study, the genotypes under investigation had the lowest grain yields in both locations due to the highest disease pressure, which is defined as foliar tissue infection. This may result from the pathogen’s hypersensitive lesions covering a portion of the leaf’s photosynthetic region. Although additional research is necessary to validate our findings, we advise breeders to adopt the genotype of Baco Striga sorghum as a check line in their breeding program to increase resistance to anthracnose disease. This recommendation is based on the results of our two locations.

In the current investigation, the disease manifested itself in a distinct way on the examined sorghum genotypes in each of the two experimental study locations. Marley et al. (2001), Thakur et al. (2007), and Tsedaley et al. (2023) have also reported findings that are similar. The degree of host susceptibility, the pathogen’s variability, and the local weather in the research regions could all play a significant role. Inoculum density, the pathogenicity of the strains, and cultural techniques also had an impact ( Pande et al. 1991; Chala et al. 2010a, b). The current study’s findings indicate that during the 2020 main cropping season, Assosa district experienced a higher severity of sorghum anthracnose than Bambasi district. This might be the result of ideal weather during that cropping session, which included heavy rainfall and high relative humidity in the Assosa district. According to Frederiksen (2000), prolonged cloudy, warm, humid, and wet weather is particularly conducive to the disease’s severity, particularly when it happens during the crop’s early grain-filling stage. The current study found a considerable negative association between AUDPC and yield, yield-related characteristics, and agronomic variables. This is consistent with Desalegn et al.’s findings ( 2022). The Assosa district showed reduced TSW, higher grain weight losses, and shorter panicle length in the current study. This is because all sorghum genotypes at that site had the highest anthracnose severity effect on record. According to Casela and Ferreira (1987), C. sublineolum conidia impede the flow of nutrients and water through the vascular tissues of the plant, which stunts the growth of the panicle and grain. Ali et al. (1987) also documented reductions in grain yield because of fungal infection, which is typically linked to a decrease in grain size.

Generally speaking, developing resistant cultivars is the main strategy for controlling sorghum anthracnose. Also, determining the ideal sowing time requires an understanding of the temporal and spatial dynamics of the disease. This understanding helps to establish management techniques that are efficient, economical, secure, and long-lasting ( Chala et al. 2010a). To consider sowing date as a management option for anthracnose, however, one must first determine the disease’s life cycle and the best time to reach its peak ( Tsedaley et al., 2023). The differences between genotypes with varied resistance levels and meteorological factors in terms of when anthracnose disease develops was also demonstrated by our most recent studies. There were notable differences in the course of the disease between the studied sorghum genotypes in each location. In the Assosa district, anthracnose development progressed more slowly on the sorghum genotypes Mersa-1, SC748-5, and Geremew; in the Bambasi district, it progressed more slowly on the sorghum genotypes ACC-BRC-18, ACC-BRC-5, and BRC-245. In both locations, the Mersa-1 genotype consistently displayed the lowest disease pressure and higher grain yields. As a result, we suggested that farmers in the districts of Assosa and Bambasi give the Mersa-1genetype some thought as the ideal substitute to fend against anthracnose and increase output in these regions. We discovered that the months of September through October were the busiest for the establishment of anthracnose disease and had a significant detrimental impact on the grain yields of Western Ethiopian sorghum genotypes. According to Chala et al. (2010a) and Tsedaley et al. (2023), the disease peaks in south and southwest Ethiopia was during August and September, respectively. This suggests that in south, southwest, and western Ethiopia, September is a more favorable month for the development of anthracnose disease. This may have to do with the increased rainfall in these regions, which creates ideal climatic conditions for the pathogen’s growth, the crop’s developmental (flowering) stage, and the pathogen’s peak period. According to Tsedaley et al. (2023), it is crucial and more successful to implement management strategies that coincide with the sensitive stage of the crop and the period when the disease develops in order to lessen the severity of the disease and increase grain yield. Moreover, adjusting the sowing date may be a significant strategy for decreasing the severity of sorghum anthracnose and ultimately raising grain yield. The findings of ( Gwary et al. 2008; Chala and Tronsmo 2011; Chala et al. 2010b) are consistent with our findings.

It is well known that three factors must coincide for any disease to develop: the host must be sensitive, the virus must be virulent, and the weather must be favorable ( Tsedaley et al. 2023). Accordingly, during the main cropping season of 2020 in Assosa district as opposed to Bambasi district, the weather is more conducive to the development of anthracnose disease on genotypes of sorghum that have been studied in Western Ethiopian fields. Under field conditions, weather factors significantly influenced the development of anthracnose disease and the grain yield of several genotypes of sorghum. The incidence and severity of sorghum anthracnose have been demonstrated to be significantly influenced by meteorological factors, including relative humidity and rainfall. Important variables for the development of anthracnose disease include relative humidity, cumulative and frequency of rainfall ( Frederiksen 1986, 2000; Erpelding and Prom 2004; Rana et al. 2020). Anthracnose is a polycyclic disease that is favorably affected by high humidity and rainfall, moderate temperatures, and a significant amount of inoculum ( Frederiksen 1986). The grain yield of every investigated sorghum genotype in both locations was negatively impacted, albeit not significantly, by the anthracnose severity assessments from the first to the fifth round. This might be because the lower leaves contribute less to the process of grain-filling, which turns photosynthetic products into grain. The current study’s findings are consistent with those of Tsedaley et al. (2023), who reported that the upper leaves play a more significant role in grain growth and that greater severity during the grain-filling to maturity stage led to a significant drop in grain production. The flag leaf ( Stoy 1963; Stamp and Herzog 1976) contributes the majority of photosynthate used in grain filling. The number of seeds per panicle increased as panicle length increased, and vice versa for grain size and grain yield. There was also a substantial positive correlation between panicle length, TSW, and grain yield. Severe anthracnose infections, meteorological conditions that affect photosynthesis and the quantity of food stored all have a significant impact on grain size ( Tsedaley et al. 2023). Thus, choosing a more resistant sorghum genotype and a suitable site may be crucial to reducing the severity of anthracnose and raising the grain yield of the sorghum crop in the Western Ethiopian districts of Assosa and Bambasi.

## Conclusion and recommendation

Rainfall, mean maximum temperature, relative humidity, and the various resistance levels of sorghum genotypes all had a significant impact on the severity of anthracnose under field conditions. Grain filling was significantly impacted negatively by disease pressure, which causes significant yield losses in genotypes that are sensitive. Therefore, managing this serious sorghum disease worldwide may benefit greatly from an awareness of these factors. The results also demonstrated that Ethiopian germplasms are very probable sources of anthracnose disease resistance genes. Farmers in the Assosa and Bambasi districts, as well as other places in Western Ethiopia with a similar agro-ecology, are advised to utilize Mersa-1 sorghum genotypes due to their good performance, which includes strong resistance to anthracnose, vigor growth, and better grain yield. Breeders might utilize the Baco Striga sorghum genotype as a check line in a breeding effort to resist anthracnose disease because it shown a high vulnerability to the disease at both locations.

## Ethics approval and consent to participate

Not applicable.

## Consent for publication

Not applicable.

## Authors’ contributions

Binyam Tsedaley and Kumlachew Alemu: designed the study, performed the experiments, analysed data and wrote the manuscript. Both authors read and approved the final manuscript.

## Data Availability

The data supporting this research are available in figshare ( Tsedaley and Alemu, 2024): **Title:** Evaluate reaction of sorghum genotypes for sorghum anthracnose in Ethiopia. **DOI**: https://doi.org/10.6084/m9.figshare.25930312.v3 **Authored by**: Binyam Tsedaley and Kumlachew Alemu Data are available under the terms of the Creative Commons Attribution 4.0 International license (CC-BY 4.0).

## References

[ref2] Ali MEK Warren HL Latin RX : Relationship between anthracnose leaf blight and losses in grain yield of sorghum. *Plant Dis.* 1987;71:803–806. 10.1094/PD-71-0803

[ref3] Bressan W Figueiredo JEF : Biological control of breeds and isolates of *Colletotrichum graminicola.* *Sorghum by Actinomycetes.* Embrapa corn and sorghum Technical Communication;2003;62.

[ref4] Cardwell KF Hepperly PR Frederiksen RA : Pathotypes of *Colletotrichum graminicola* and seed transmission of sorghum anthracnose. *Plant Dis.* 1989;73:255–257. 10.1094/PD-73-0255

[ref5] Casela CR Ferreira AS : Proposal of a system for classifying races of *Colletotrichum graminicola*, causal agent of anthracnose in sorghum ( *Sorghum bicolor*) (Ces.) Wils in Brazil. *Fitopatol Brazil.* 1987;12:337–344.

[ref6] Central Statistical Agency (CSA): The Federal Democratic Republic of Ethiopia Agricultural Sample Survey 2011/2012 (2004 E.C.). Volume I, Report on Area and Production of Major Crops, (Private Peasant Holdings, Meher Season). 2017.

[ref7] Chala A Alemu T Prom LK : Effect of host genotypes and weather variables on the severity and temporal dynamics of sorghum anthracnose in Ethiopia. *Plant Pathol. J.* 2010b;9:39–46. 10.3923/ppj.2010.39.46

[ref8] Chala A Brurberg MB Tronsmo AM : Prevalence and intensity of sorghum anthracnose in Ethiopia. *J. SAT Agric. Res.* 2007;5:1–3.

[ref9] Chala A Brurberg MB Tronsmo AM : Incidence and severity of sorghum anthracnose in Ethiopia. *Plant Pathol. J.* 2010a;9:23–30. 10.3923/ppj.2010.23.30

[ref10] Chala A Tronsmo AM : Evaluation of Ethiopian sorghum accessions for resistance against *Colletotrichum sublineolum.* *Eur. J. Plant Pathol.* 2011;132:179–189.

[ref12] Chaube HS Pundhir VS : *Crop disease and their Management.* New Delhi: Prentice Hall of India;2005;703.

[ref14] Da Costa RV Casela CR Zambolim L : The sorghum anthracnose. *Brazilian.* *Phytopathology.* 2003;28:345–354.

[ref15] Dessalegn K Lule D Nida H : Evaluation of selected Ethiopian sorghum genotypes for resistance to anthracnose. *Eur. J. Plant Pathol.* 2022;162:79–91. 10.1007/s10658-021-02386-6

[ref16] Erpelding JE : Anthracnose Disease Response for Photoperiod-Insensitive Ethiopian Germplasm from the U.S. Sorghum Collection. *World J. Agric. Sci.* 2009;5(6):707–713.

[ref17] Erpelding JE Prom LK : Evaluation of Malian sorghum germplasm for resistance against anthracnose. *Plant Pathol. J.* 2004;3:65–71.

[ref18] Fetene M Okori P Gudu S : *Delivering New Sorghum and Finger Millet Innovations for Food Security and Improving Livelihoods in Eastern Africa.* International Livestock Research Institute (ILRI);2011.

[ref19] Food and Agriculture Organization (FAO): Food and agriculture data. 2021.

[ref20] Frederiksen RA : Leaf Anthracnose. *Compendium of Sorghum Diseases.* St. Paul, MN: The American Phytopathological Society;2000; pp.10–12.

[ref21] Frederiksen S : Revision of taeniatherum ( *Poaceae*). *Nord. J. Bot.* 1986;6:389–397. 10.1111/j.1756-1051.1986.tb00894.x

[ref22] Girma T Fekede A Temam H : Review of Maize, Sorghum and Millet Pathology Research. Tadesse A , editors. *Increasing Crop Production through Improved Plant Protection-Volume II. Plant Protection Society of Ethiopia (PPSE).* Ethiopia: PPSE and EIAR, Addis Ababa;2009; pp.245–301.

[ref23] Gwary DM Asala SW : Cost-benefit of fungicidal control of anthracnose on sorghum in Northern Nigeria. *Int. J. Agric. Biol.* 2006;8(3):306–308.

[ref24] Gwary DM Bwatanglang N Bdliya BS : Integrated Management of Sorghum Anthracnose Through the Use of Fungicides, Crop Varieties and Manipulation of Sowing Dates in Sudan Savanna of Nigeria. *Int. J. Agric. Biol.* 2008;10:661–664.

[ref62] Khan MF Ahmad I Ali S : Biocontrol agents for fungal pathogens: Mechanisms and effectiveness. *Biol. Control.* 2018;121:86–94.

[ref25] Marley PS Thakur RP Ajayi O : Variation among foliar isolates of *Colletotrichum sublineolum* of sorghum in Nigeria. *Field Crop Res.* 2001;69:133–142. 10.1016/S0378-4290(00)00128-3

[ref26] Marley PS Thakur RP Ajayi O : Variation among foliar isolates of *Colletotrichum sublineolum* of sorghum in Nigeria. *Field Crop Res.* 2004;69:133–142.

[ref27] Mekbib F : Farmer and formal breeding of sorghum ( *Sorghum bicolor* (L.) Moench) and the implications for integrated plant breeding. *Euphytica.* 2006;152:163–176. 10.1007/s10681-006-9191-7

[ref28] Mengistuc G Lule D Desalegn K : Registration of “Chemeda and Gemedi” Sorghum Varieties. *East Afr. J. Sci.* 2013;7:61–62.

[ref31] Ngugi HK Julianb AM Kinga SB : Epidemiology of sorghum anthracnose ( *Colletotrichum sublineolum*) and leaf blight ( *Exserohilum turcicum*) in Kenya. *Plant Pathol.* 2000;49:129–140. 10.1046/j.1365-3059.2000.00424.x

[ref32] Pande S Mughogho LK Badhiopadhyay R : Variation in pathogenicity and cultural characteristics of sorghum isolates of *Colletotrichum graminicola* in India. *Plant Dis.* 1991;75:778–783. 10.1094/PD-75-0778

[ref60] Pande S Bandyopadhyay R BlüMmel M : Disease management factors influencing yield and quality of sorghum and groundnut crop residues. *Field Crop Res.* 2003;84(1–2):89–103.

[ref33] Pastor-Corrales MA Frederiksen RA : Sorghum anthracnose. *Sorghum Diseases: A World Review. Proc. In Workshop Sorghum Dis.* Williams RJ Frederiksen RA Mughogho LK , editors. Patancheru, India: International Crops Research Institute for the Semi-Arid Tropics;1980; pp.289–294.

[ref34] Pedersen JF Marx DB Funnell DL Use of A _3_ cytoplasm to reduce risk of gene flow through sorghum pollen. *Crop Sci.* 2003;43:1506–1509. 10.2135/cropsci2003.1506

[ref35] Prom LK Perumal R Erpelding J : A Pictorial Technique for Mass Screening of Sorghum Germplasm for Anthracnose ( *Colletotrichum sublineolum*) Resistance. *Open Agric. J.* 2009;3:20–25. 10.2174/1874331500903010020

[ref36] Rana M Singh Y Srivastava S : In vivo evaluation of fungicides and biocontrol agents against anthracnose of sorghum. *Pl Cell Biotechnol. Mol. Biol.* 2020;21:8–14.

[ref37] Rana M Singh Y Srivastava S : Effect of Weather Parameters on the Severity of Sorghum Anthracnose. *Environ. Ecol.* 2021;39(4A):1236–1241. 0970-0420.

[ref38] Sahile S Fininsa C Sakhuja PK : Yield loss of faba bean ( *Vicia faba*) due to chocolate spot ( *Botrtis faba*) in sole and mixed cropping system in Ethiopia. *Arch. Phytopathol. Plant Protect.* 2008;43(12):1144–1159.

[ref39] SAS. Inc: *Statistical Analysis Software (SAS) Guide for Personal Computers. Version 9.2.* Cary, NC: SAS Institute;2015.

[ref40] Shaner G Finney R : Inheritance of slow mildewing resistance in wheat. *Proc. Am. Phytopatol.* 1977;28:105–112.

[ref61] Sharma P Sharma S Singh M : Role of antagonistic microorganisms in managing plant diseases. *J. Agric. Sci. Technol.* 2019;19(6):1571–1583.

[ref41] Smith CW Frederiksen RA : *Sorghum: Origin, History, Technology and Production.* New York: John Wiley and Sons;2000.

[ref42] Stamp P Herzog H : Flag-leaf senescence and grain growth in certain German varieties of spring wheat ( *Triticum aestivum* L.). *Z Pflanzenzucht.* 1976;77:330–338.

[ref43] Stoy V : The translocation of C ^14^-labelled photosynthetic products from the leaf to the ear in wheat. *Physiol. Plant.* 1963;16(4):851–866. 10.1111/j.1399-3054.1963.tb08363.x

[ref44] Sutton BC : The Coelomycetes: Fungi Imperfecti with Pycnidia, Acervuli, and Stromata. *Commonwealth Mycological Institute, Kew, London Microbiology.* 1980;74:823–832. 10.1079/9780851984469.0000

[ref45] Tesso T Perumal R Little CR : Sorghum Pathology and Biotechnology- A Fungal Disease Perspective: Part II. Anthracnose, Stalk Rot, and Downy Mildew. *Eur. J. Plant Sci. Biotechnol.* Global Science Books.2012.

[ref46] Thakur RP : Anthracnose. Thakur RP Reddy BVS Mathur K , editors. *Screening techniques for sorghum diseases. Information Bulletin No. 76.* Andhra Pradesh: ICRISAT;2007; pp.53–57.

[ref47] Thakur RP Mathur K : Anthracnose. *Compendium of Sorghum Diseases.* Frederiksen RA Odvody GN , editors. St. Paul, MN: American Phytopathological Society;2000; pp.10–12

[ref48] Thakur RP Reddy BV Mathur K : *Screening Techniques for Sorghum Diseases. Information Bulletin No. 76.* Patancheru 502 324, Andhra Pradesh, India: International Crops Research Institute for the Semi-Arid Tropics;2007;92.

[ref49] Thomas MD : Sorghum anthracnose research in West Africa: A look at the present and future. Leslie JF Frederiksen RA , editors. *Disease Analysis Through Genetics and Biotechnology: Interdisciplinary Bridges to Improved Sorghum and Millet Crops.* Ames, Iowa: Iowa State University Press;1995; pp.127–136.

[ref50] Tsedaley B Adugna G Lemessa F : Distribution and importance of sorghum Anthracnose ( *Colletotrichum sublineolum*) in southwestern and western Ethiopia. *Plant Pathol. J.* 2016;15(3):75–85. 10.3923/ppj.2016.75.85

[ref51] Tsedaley B Adugna G Lemessa F : *Evaluation of Sorghum Genotypes and Influence of Weather Variables on Anthracnose (Colletotrichum sublineolum) Disease Development under Field Conditions at Jimma.* Southwestern Ethiopia: HELIYON;2023. 10.1016/j.heliyon.2023.e15297 PMC10130866 37123970

[ref52] Tsedaley B Adugna G Lemessa F : *Pathogenic variability of Colletotrichum sublineolum isolates on sorghum differentials under greenhouse conditions in Jimma.* Ethiopia: Archives of Phytopathology and Plant Protection;2021. 10.1080/03235408.2021.1988

[ref53] Tsedaley B Alemu K : Evaluate reaction of sorghum genotypes for sorghum anthracnose in Ethiopia.Dataset. *figshare.* 2024. 10.6084/m9.figshare.25930312.v3

[ref54] United State Department of Agriculture (USAD): USAID Office of Food for Peace Ethiopia, Bellmon Estimation, Annex 1 Economic Data and Trends. 2012 September.

[ref55] Upadhyaya HD Wang YH Sharma R : Identification of genetic markers linked to anthracnose resistance in sorghum using association analysis. *Theor. Appl. Genet.* 2013;126:1649–1657. 10.1007/s00122-013-2081-1 23463493

[ref56] Van der Plank JE : *Plant Disease: Epidemics and Control.* Academic Press;1963.

[ref57] Valerio HM Resende MA Weikert-Oliveira RCB : Virulence and molecular diversity in *Colletotrichum graminicola* from Brazil. *Mycopathologia.* 2005;159:449–459. 10.1007/s11046-005-0373-y 15883732

[ref58] Wheeler BJ : *An Introduction to Plant Diseases.* John Wiley and Sons, Ltd;1969.

[ref59] Wortmann C Mamo M Mburu C : Atlas of Sorghum ( *Sorghum bicolor* (L.): Moench): Production in Eastern and southern Africa. International sorghum and Millet Collaboration Research Support Program (INTSORMIL CRSP). 2009.

