# Supplementary material for: Temporal and Spatial Dynamics of Anthracnose ( Colletotrichum sublineolum) Disease on Selected Sorghum Genotypes at Assosa Zone, Western Ethiopia
Source: F1000Res. 2025 Mar 17;13:1290. Originally published 2024 Oct 28. [Version 2] doi: 10.12688/f1000research.156145.2 (PMC12149804; doi:10.12688/f1000research.156145.2)
Supplement: Peer review history

# Reviewer report: Reviewer response for version 2

**DOI:** 10.5256/f1000research.178969.r381619

**Published:** 2025-06-09

**Authors:** Temesgen Zewotir

**Affiliations:** 1 University of KwaZulu Natal, KwaZulu-Natal, South Africa

The paper is well motivated and  well written.  The data and the analysis is well  executed.  The analysis provide excellent insight about  disease progress rate, yield-related trait, sorghum grain yield, and mean severity index at the two research stations: the Bambasi district and  Assosa district. Occasionally the authors tend to compare and draw firm conclusion  between the two stations results without statistical significance test. Describing the two station results is perfectly acceptable but comparing drawing a conclusion should be done with appropriate statistical test. Accordingly I suggest the authors to be cautious of this.  Secondly the authors indicated they used SAS software for their analysis, it would be useful to state which SAS procedure they have used for each analysis..

Is the work clearly and accurately presented and does it cite the current literature?

Yes

If applicable, is the statistical analysis and its interpretation appropriate?

Partly

Are all the source data underlying the results available to ensure full reproducibility?

Partly

Is the study design appropriate and is the work technically sound?

Yes

Are the conclusions drawn adequately supported by the results?

Yes

Are sufficient details of methods and analysis provided to allow replication by others?

Yes

Reviewer Expertise:

Statistics

I confirm that I have read this submission and believe that I have an appropriate level of expertise to confirm that it is of an acceptable scientific standard.

# Reviewer report: Reviewer response for version 2

**DOI:** 10.5256/f1000research.178969.r371384

**Published:** 2025-03-24

**Authors:** Ararsa Leta

**Affiliations:** 1 Ambo University, Ambo, Oromia, Ethiopia

I appreciate the authors' efforts in addressing the reviewer comments and making substantial improvements to the manuscript. The incorporation of additional data in Table 1, the refinements in text clarity, and the adjustments to table numbering have significantly enhanced the overall quality and readability of the study. The revisions effectively strengthen the manuscript’s conclusions and provide a clearer presentation of the findings.

 Based on these improvements, I find the revisions to be appropriate and sufficient.

Is the work clearly and accurately presented and does it cite the current literature?

Yes

If applicable, is the statistical analysis and its interpretation appropriate?

Yes

Are all the source data underlying the results available to ensure full reproducibility?

Yes

Is the study design appropriate and is the work technically sound?

Yes

Are the conclusions drawn adequately supported by the results?

Yes

Are sufficient details of methods and analysis provided to allow replication by others?

Yes

Reviewer Expertise:

NA

I confirm that I have read this submission and believe that I have an appropriate level of expertise to confirm that it is of an acceptable scientific standard.

# Reviewer report: Reviewer response for version 1

**DOI:** 10.5256/f1000research.171415.r366819

**Published:** 2025-03-03

**Authors:** Ararsa Leta

**Affiliations:** 1 Ambo University, Ambo, Oromia, Ethiopia

Introduction

1. paragraph 1, the last citation. (USAD 2012) Do You mean USAID? Please check.
2. paragraph 2, the last lines. The term foliar;  The term is not appropriate to indicate the phonological growth stage of the crop; it is simply the above ground plant part at different stages. Do you mean vegetative stage? Please correctly indicate .
3. paragraph 4, 3 ^rd^ line. The application of biocontrol methods has been suggested by Michereff et al. (1994) as a potential remedy for anthracnose management. Too old to reflect the current advancement and reality of the potential of biocontrol method for this pathogen. Please replace with recent one.

Materials and methods

1. In materials for the experiment.  Sartu, ACC-BRC-18, BRC-378, ACC-BRC-5, BRC-245, BTx623, SC748-5, Assosa-1, and Mersa-1. It is good if there is a table for detail description of the genotypes including their year of release, center released the varieties, source of the material and anthracnose reaction at time of release.

Results

1. In the 1 ^st^ paragraph last lines. This showed that the seven …. Why seven? While you evaluate ten genotype? What is you reference point to say genotype show disease reduction?
2. **In section Sorghum genotypes' agronomic characteristics. ** Delete the phrase ‘In the 2020 cropping season’, Since your data is one year data no need to mention the year hear, and you already mentioned the experiment is done in 2020 in methodology
3. **In section Components of sorghum genotypes related to yield. **Delete the phrase ‘In the 2020 cropping season’
4. **In section Sorghum genotypes' grain yields.** Delete the phrase ‘In the 2020 cropping season’
5. **In section Sorghum genotypes' grain yields 2 ^nd^ paragraph. **The ten frequently…. Delete term frequently, the genotypes are once evaluated in the current study **, **The nine sorghum genotypes'….. Delete frequently. The genotypes are once evaluated in the current study
6. **Table 4.** The correlation coefficient between weather variables and the sorghum anthracnose severity (PSI) at two districts in Western Ethiopia, Assosa and Bambasi, was measured at weekly intervals. - As the weather variables for the two districts were different please do the analysis separately and present the table for each district

Is the work clearly and accurately presented and does it cite the current literature?

Yes

If applicable, is the statistical analysis and its interpretation appropriate?

Yes

Are all the source data underlying the results available to ensure full reproducibility?

Yes

Is the study design appropriate and is the work technically sound?

Yes

Are the conclusions drawn adequately supported by the results?

Yes

Are sufficient details of methods and analysis provided to allow replication by others?

Yes

Reviewer Expertise:

NA

I confirm that I have read this submission and believe that I have an appropriate level of expertise to confirm that it is of an acceptable scientific standard, however I have significant reservations, as outlined above.

# Response

**Published:** 2025-03-04

**Authors:** Binyam Tsedaley

**Affiliations:** Plant Science, Assosa University, Asosa, Benishangul-Gumuz, Ethiopia

**Response to Reviewer**

**Introduction**

1. **Paragraph 1, last citation (USAD 2012).** Do you mean USAID? Please check.
  ✔ Yes, it was a typographical error. The correct citation is **USAID (United States Agency for International Development)**, and we have corrected it accordingly.
2. **Paragraph 2, last lines.** The term *foliar* is not appropriate for indicating the phonological growth stage of the crop; it refers to above-ground plant parts at different stages. Did you mean *vegetative stage*? Please clarify.
  ✔ Yes, this was a typographical error. The correct term is **flowering stage**, not *foliar stage*. We have revised it accordingly.
3. **Paragraph 4, third line.** The application of biocontrol methods was suggested by Michereff et al. (1994) for anthracnose management. This reference is outdated and does not reflect current advancements in biocontrol methods. Please replace it with a more recent source.
  ✔ We agree and have replaced the reference with the following updated statement:

"Biological control using antagonistic organisms such as *Trichoderma* species, *Pseudomonas syringae*, and *Pseudomonas chlororaphis* has been extensively studied due to their characteristics, including fast competition, parasitism, plant root colonization, induced systemic resistance, plant growth promotion, and production of antibiosis (Khan et al., 2018; Sharma et al., 2019)."

**Materials and Methods**

1. **Description of sorghum genotypes:** It would be beneficial to provide a table detailing the genotypes, including the year of release, the center that released them, their source, and their anthracnose reaction at the time of release.
  ✔ We appreciate this suggestion and have now included the following table in our manuscript:

**Table  1. Sorghum varieties with their crop passport**

 S.N

 Variety name

 Pedigree Name

 Year of Release

 Disease Reaction

 Altitude

 Yield (q/ha)

 Seed Source

 Research Center

 Farmers Field

 1

 Aba-Melko

 Sartu

 2001

 R

 75

 50

 JARC

 2

 Chemeda

 ACC-BRC-18

 2013

 MR

 1500-1900

 32

 25

 BARC

 3

 Dano

 BRC-378

 2006

 MR

 1500-1900

 40-50

 30-48

 BARC

 4

 Gemedi

 ACC-BRC-5

 2013

 MR

 1500-1900

 33

 28

 BARC

 5

 Lalo           

 BRC-245

 2006

 MR

 1500-1900

 40-52

 35-48

 BARC

 6

 Universally Susceptible

 BTX623

 -

 HS

 -

 -

 -

 MARC

 7

 -

 PI570726

 -

 HR ^*^

 -

 -

 -

 MARC

 8

 -

 SC748-5

 -

 HR ^*^

 -

 -

 MARC

 9

 Assosa-1

 -

 Under seed multiplication

 -

 -

 -

 -

 Assosa University

 10

 Local Chuck

 -

 -

 -

 -

 -

 -

 -

- Source: MOA and BARC         
- Where: HR*= Highly Resistant under greenhouse, HS= Highly Susceptible, MR =Moderately Resistant, R= Resistant, S = Susceptible and - = no information

**Results**

1. **First paragraph, last lines:** The phrase *"This showed that the seven…"* – Why mention *seven* genotypes when ten were evaluated? What is the reference point for identifying genotypes with disease reduction?
  ✔ As indicated in **Table 2**, only seven genotypes ( *excluding Baco Striga, Mersa-1, and SC748-5*) showed severity reduction at Bambasi compared to Assosa district. This is why we referred to seven genotypes rather than ten.
2. **Section: Sorghum genotypes' agronomic characteristics.** Delete the phrase *"In the 2020 cropping season."* Since the study covers only one year, this phrase is unnecessary. The methodology section already mentions that the experiment was conducted in 2020.
  ✔ We accept the comment and have deleted *"In the 2020 cropping season"* from the manuscript.

 

1. **Section: Components of sorghum genotypes related to yield. Delete the phrase *"In the 2020 cropping season."***
** ✔ We have removed this phrase as suggested.**
2. **Section: Sorghum genotypes' grain yields. Delete the phrase *"In the 2020 cropping season."***
** ✔ We have deleted the phrase from the manuscript.**
3. **Section: Sorghum genotypes' grain yields (second paragraph). The phrase *"The ten frequently evaluated genotypes…"* should be revised. The term *"frequently"* is misleading because the genotypes were evaluated only once in this study.**
** ✔ We agree and have removed the word *"frequently"*, revising the sentence accordingly.**
4. **Table 4: Correlation between weather variables and anthracnose severity (PSI) at two districts in Western Ethiopia, Assosa and Bambasi. The weather data for the two districts are different. Please analyze and present them separately.**
** ✔ We appreciate the suggestion. However, since weather data was only available for Assosa district and not for Bambasi district, a separate analysis is not possible. Instead, we have revised the table title to specify that the correlation analysis pertains only to Assosa district.**

Regarding the term *"frequently tested genotypes"*, it was intended to indicate that these six sorghum genotypes were evaluated over two cropping seasons at Jimma.

 This revised response is clear, concise, and professional, addressing all reviewer comments effectively. Let me know if you need any further refinements!
